# Effects of Route Complexity and Lighting on Route Following in Alzheimer’s Disease and Posterior Cortical Atrophy

**DOI:** 10.3390/brainsci14121217

**Published:** 2024-11-30

**Authors:** Amelia M. Carton, Chris Frost, Teresa Poole, Biao Yang, Ian D. McCarthy, Tatsuto Suzuki, Catherine Holloway, Robin Serougne, Derrick Boampong, Mary Pat Sullivan, Nick Tyler, Sebastian Crutch, Keir X. X. Yong

**Affiliations:** 1 Dementia Research Centre, Department of Neurodegeneration, UCL Queen Square Institute of Neurology, University College London, London WC1N 3BG, UK; amelia.carton@gstt.nhs.uk (A.M.C.);; 2 Evelina London Children’s Hospital, Guys & St Thomas’ NHS Foundation Trust, London SE1 7EH, UK; 3 Department of Medical Statistics, Faculty of Epidemiology and Population Health, London School of Hygiene & Tropical Medicine, London WC1E 7HT, UK; chris.frost@lshtm.ac.uk (C.F.);; 4 Pedestrian Accessibility Movement Environment Laboratory, Department of Civil, Environmental and Geomatic Engineering, University College London, London WC1E 6BT, UK; 5 Faculty of Education and Professional Studies, Nipissing University, North Bay, ON P1B 8L7, Canada

**Keywords:** Alzheimer’s disease, built environment, dementia, accessibility, inertial measurement units, navigation, gait

## Abstract

Objective: Visual processing deficits arising in dementia are associated with particular functional disability. This study aimed to investigate the effects of the built environment on mobility and navigation in people with dementia-related visual loss. Methods: Participants with posterior cortical atrophy (PCA; “visual-variant Alzheimer’s”; n = 11), typical Alzheimer’s disease (tAD; N = 10), and controls (n = 13) repeatedly walked down routes within a simplified real-world setting. Participant groups were of comparable age and gender. Routes were of different complexity (straight, U-shaped, and S-shaped), overhead lighting levels (low and high) and with or without a dynamic LED (light-emitting diode) cue (trial n = 24). Ratios of walking times for each experimental condition (each complex route vs the straight route, high lighting vs low, and LED cue vs no cue) were compared between participant groups. Kinematic measures were produced from a total of 10,813 steps using wearable inertial measurement units (IMUs). Results: The walking time ratios relating to route complexity were higher in the PCA group than in controls: 30.3% (95% CI [13.5%, 49.5%] higher for U-shaped vs straight and 31.9% [21.1%, 55.3%] for S-shaped vs straight, averaged over other conditions). The analogous results relating to route complexity for the tAD group were intermediate between those for the PCA and control groups. There was no evidence that walking time ratios differed according to lighting level or the presence of the LED cue. Conclusions: Findings contribute to evidence-based design for dementia-friendly environments, emphasizing consequences of environmental complexity for functional independence and mobility in people with dementia-related visual loss. Findings inform recommendations for environmental design to support the independence of individuals with dementia.

## 1. Introduction

Alongside prominent impairments in episodic memory, people with Alzheimer’s disease experience significant visual processing deficits consistent with the neurodegeneration of posterior brain regions [1,2]. Such deficits include reduced depth perception, reduced sensitivity for low spatial frequencies, a constricted window of spatial attention, and elevated optic flow thresholds [3,4,5,6]. These deficits may contribute to characteristic behaviors associated with typical, amnestic AD (tAD; e.g., becoming lost or difficulties locating objects). Impaired visual processing becomes more prominent over the disease’s course [7,8], carrying considerable implications for daily functioning.

Posterior cortical atrophy (PCA) provides a unique opportunity to investigate the impact of early and predominant visual processing impairment arising from neurodegenerative disease. PCA is a syndrome characterized by progressive visual impairment, with memory, language, and insight remaining relatively preserved [9,10,11]. Consistent with early visuoperceptual and visuospatial loss, PCA tends to initially affect parietal, occipital, and occipitotemporal regions of the brain [7]. PCA is most frequently caused by Alzheimer’s disease pathology; although, other underlying causes can include dementia with Lewy bodies, corticobasal degeneration, or prion disease [10].

Visual processing deficits pose particular challenges to independence for people with dementia. There is evidence that visuospatial dysfunction may be more strongly associated with functional abilities for individuals with tAD relative to episodic, semantic, and working memory, attention, and executive function [12]. For individuals living with PCA, these visuospatial and visuoperceptual impairments likely relate to difficulties with driving, getting lost, and environmental disorientation [13,14]. Such loss of functional status renders individuals with PCA disproportionately reliant on their caregivers despite relatively spared cognitive abilities and insight. Visual processing impairment in dementia has consequences for safety and is associated with a risk of falling and difficulties with self-care [15,16].

The physical environment may play an elevated role in mediating functional independence for people with dementia, especially those exhibiting visual processing deficits. Environmental guidelines emphasize the importance of adapting the physical environments to maximize the independence of people with dementia (e.g., lighting, signage, and color contrast). Designing environments specifically for people with dementia may promote their independence and quality of life by compensating for their specific disabilities. However, there is a relatively limited body of quantitative literature to support current advice and design guidelines, leading to calls for further empirical research [17,18].

Corridors may be particularly important to consider when designing environments for people with dementia. Navigational difficulties and spatial disorientation are early symptoms of tAD and, especially, PCA [19,20]. However, there is limited consensus regarding what constitutes a golden standard of dementia design in these areas. Previous studies suggested ‘L-shaped’ corridors were preferential for people with dementia to navigate compared to straight corridors or square or H-shaped corridors [21]. However, subsequent investigations have suggested that straight or circular corridors may mitigate disorientation or wandering behaviors by limiting decision points [22,23].

Beyond the structural design of the built environment, visually salient cues may support aspects of functional independence in people with dementia. Strategic use of visual contrast may facilitate food and liquid intake and wayfinding [15,24]. There is evidence that contrast-based cues may support visually guided navigation in PCA and tAD [14]. Visual motion may offer a promising cue given that people with PCA have been reported as being better able to locate moving, rather than stationary, objects [25,26]. Lighting moderates contrast and saliency and is often considered an important environmental aspect to consider when designing environments for people with dementia [22]. Higher lighting levels are often recommended to enable people to adequately perceive the environment, faces, and signs and to reduce falls for people with dementia [27]. However, contradictory and contentious recommendations and low-quality evidence have been noted regarding environmental design for people with dementia and particularly those with loss of sight [18,22].

The current investigation comprises tasks that involve navigating routes within the Pedestrian Accessibility Movement Environment Laboratory (PAMELA), a simulated real-world environment that enables controlling aspects of the physical environment such as layout, lighting, and dimensions. This study investigated the effects of route complexity, the use of a dynamic LED cue, and overhead lighting on the navigational abilities of people with tAD, PCA, and healthy control participants using three differently shaped, closed routes. Task performance was evaluated through completion time and kinematic gait measures. We hypothesized that there would be a disproportionate decrease in the ability of patients to follow more complex routes, with people with PCA exhibiting particular difficulty due to their increased degree of visual processing impairment. Additionally, we hypothesized that a dynamic cue and higher levels of overhead lighting would facilitate task performance, with improvements from both being particularly evident in participants with a greater degree of visual impairment.

## 2. Materials and Methods

### 2.1. Participants

A total of 11 people with PCA, 10 people with tAD, and 13 healthy controls took part in this study. PCA group participants fulfilled clinical diagnostic criteria for PCA [9,28,29], and tAD group participants fulfilled criteria for typical amnestic Alzheimer’s disease [30]. Participant numbers were restricted owing to the relative rarity of PCA. Participants unable to walk without mobility aids were excluded. Groups were of comparable age (mean age ± SD: PCA: 65.2 ± 6; tAD: 66.2 ± 5; controls: 63.8 ± 4) and gender (male–female: PCA: 5:6; tAD: 4:6; controls: 7:6; see Supplementary Table S1). Molecular pathology (18F amyloid imaging performed as part of another investigation or CSF) was available for 6/11 PCA and 6/10 tAD patients; all were consistent with Alzheimer’s disease pathology.

### 2.2. Ethical Approvals and Consent

The National Research Ethics Service London-Queen Square ethics committee provided ethical approval for this study, and written, informed consent was obtained from all participants.

### 2.3. Background Neuropsychology

A battery of neuropsychological tests was administered to both PCA and tAD group participants. Individual scores on each test and normative means are shown in Supplementary Table S1.

### 2.4. Built Environment

Participants were invited to visit the PAMELA, University College London (UCL). The overall dimensions of the environment were 6 m × 8.4 m, formed by oak-colored wooden panels (2.0 m (H) × 1.2 m (W) and 0.04 m (D)), which were in high contrast to the dark-blue carpet that covered the floor of the platform. A high contrast between the walls and flooring is consistent with current recommendations on good design for residential homes for people with dementia and sight loss [31].

All routes were without obstacles, steps, or inclines. Routes varied according to lighting level, the presence of a dynamic LED visual cue, route complexity, and the direction for walking.

### 2.5. Route Complexity

**Figure 1:**
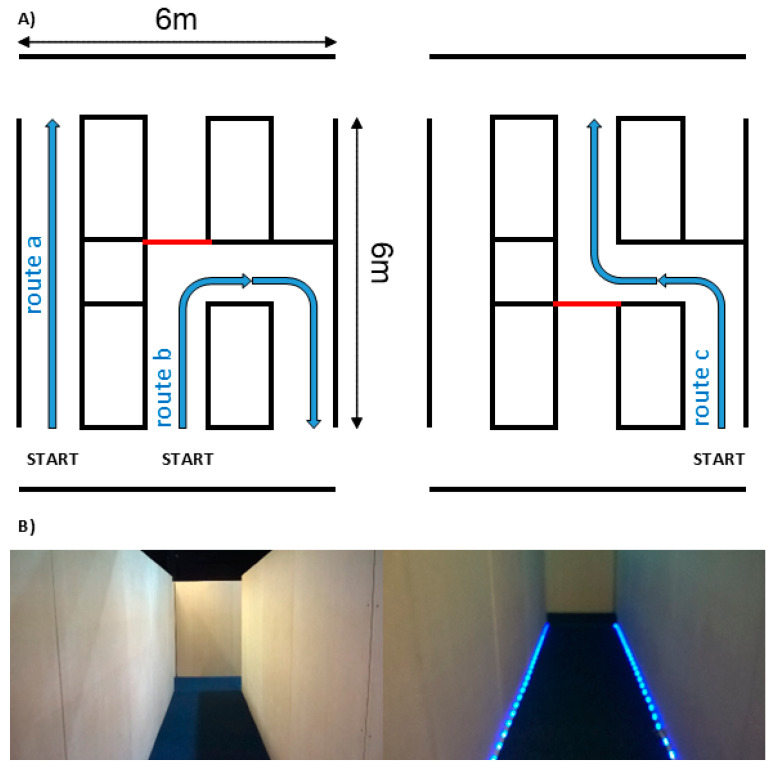
(**A**) Diagram depicting straight route, U-shaped route, and S-shaped route and (**B**) straight route without (left) and with LED cue (right).

The simulated real-world environment was manipulated to provide three different routes varying in complexity: straight (route a), U-shaped (route b), and S-shaped (route c) (see Figure 1A).

### 2.6. Lighting Level

Overhead lighting was either ‘low’ (average of 20 lux) or ‘high’ (average of 190 lux). Moreover, 50–100 lux is the typical minimum illuminance of indoor corridors specified in the guidance for lighting.

### 2.7. Dynamic LED Visual Cue

In ‘cue’ conditions, RGB (red–green–blue) LED strips were placed along either side of the corridor (Figure 1B). The LED lights along the strip simulated a ‘moving’ pattern in the direction the participant was required to walk. This ‘movement’ was created by turning two adjacent LEDs out of every six LEDs ‘ON’, keeping the remaining four ‘OFF’, which were chased by the next LEDs at a refresh rate of 50 ms. The strip consisted of 30 LEDs/m with an overall power of 9.5 W and a working voltage of DC 5V. The LEDs were programmed to appear as a moderate blue color (RGB value of 0, 0, 30). In the “no cue” condition, the LED light strip was removed.

### 2.8. Procedure and Apparatus

Participants were requested to “keep walking until you reach the end of the corridor”. These instructions were additionally repeated if participants became overtly distracted. No reference was made to the different overhead lighting conditions, the presence of the dynamic visual cue, or the route condition. Preceding each trial, the experimenter counted backwards from three before signaling the start of the trial (“Start”). All participants began each trial at the entrance of the corridor facing into the corridor, equidistant from each lateral wall. Participants had up to 60 s to reach the end of each route.

Trials were administered through a repeated-measures design with an equal numbers of levels of the following variables: route shape (straight, U-shaped, and S-shaped), LED cue (cue and no cue), lighting level (high and low), and direction (forwards and backwards). Two testing blocks were carried out comprising all 12 combinations of route, cue, and lighting level conditions (initial trial direction: block 1: forwards; block 2: backwards), for a total of 24 trials. Route conditions were counterbalanced within participants, and cue and lighting level conditions were arranged in one of four counterbalanced variants of a Latin square design; variants were assigned randomly to participants to control for order effects. In order to record a measure of gait and to produce walking paths, three wireless inertial measurement units (IMUs; Xsens MTw) recorded the movement of the pelvis and each foot at 50 Hz.

### 2.9. Data Analysis

#### 2.9.1. Completion Time

The start of each trial was manually determined as the point at which both of the participant’s feet were halfway through the first panel (0.6 m from the start of the route); the cut-off for trials was defined as 60 s after the experimenter had said “Start”. Erroneous trials were defined as when participants (1) did not reach the end of the route within the cut-off or (2) walked back to the starting point within the cut-off. All erroneous trials were censored at 60 s. Completion times were defined as the time taken between both feet passing halfway through the first panel (0.6 m from the start of the route) and both feet passing halfway through the final panel (0.6 m from the end of the route). Owing to the cue malfunctioning, one participant in the tAD group performed only the first four trials in the presence of the visual cue (forwards/backwards under straight/U-shaped and high lighting conditions).

The statistical analysis involved both within-group and between-group comparisons. In brief, within each of the three groups (PCA, tAD, and controls), we investigated the effects of each experimental condition on completion times by conducting analyses of ratios of completion times. Five such ratios (light high/low, cue/no cue, route U-shaped/straight, route S-shaped/straight, and route S-shaped/U-shaped) were considered. If the lighting and cue conditions had no impact on completion times, these ratios are expected to be equal to one. Conversely, the between-route ratios will not equal one: here, the interest is primarily on comparing these within-group ratios between participant groups. Accordingly, we also compared these within-group ratios between groups by expressing them as ratios (hence, resulting in ratios of ratios). We log-transformed completion times in our statistical analysis, back transforming to give geometric mean ratios (and ratios of ratios). This approach ensures that (for example) a doubling and a halving are regarded as opposite effects of equal magnitude (the geometric mean of 0.5 and 2 is 1, whereas their arithmetic mean is 1.25).

In detail, a two-stage approach was adopted to analyze the data in order to allow for the complex, repeated-measures structure of the data, for the censoring of some completion times at 60 s, and for the fact that the residual variance in (log-transformed) time taken to complete the task differed quite markedly between participants even within the same group. In the first stage, a separate model was fitted to the log-transformed completion time values for each participant. The model was a censored normal regression model that included the main effects of route, lighting, cue, direction, and the two-way interactions between route and lighting, between route and cue, and between lighting and cue (except for the participant with missing responses where some interaction terms were necessarily omitted). Taking appropriate linear contrasts over fitted values gave, for each participant, the estimated effects of lighting (high vs. low) and cue (cue vs. no cue) and three estimated effects of route (U-shaped vs. straight, S-shaped vs. straight, and S- vs. U-shaped), all expressed as differences in log-transformed times. In the second stage, one-sample *t*-tests were used to estimate and test the mean effects of lighting, cue, and route in each participant group. Two-sample *t*-tests (with Satterthwaite degrees of freedom to make a necessary allowance for differential variability) were then used to make comparisons between groups. All differences and 95% CIs of the log-transformed scale were exponentiated to permit interpretation of the results as ratios and ratios of ratios.

#### 2.9.2. Kinematic Assessment

IMU data were analyzed using Matlab 2020b during times corresponding to the trial period. The first step was excluded for each trial.

To obtain data for gait analysis, firstly, local IMU accelerations were converted to global coordinates using a rotation matrix, which were integrated to produce velocity. At this point, sensor drift was corrected for by identifying periods of rest via the feet sensors, then re-integrating the data to produce displacement along the x, y, and z planes [32]. This provided measures of step time and step length (control n = 2775; PCA n = 4561; tAD n = 3477; total step n = 10,813). Outlying long step times were considered hesitant steps and were detected using an iterative procedure adapted from McCarthy and colleagues [33]. For each of the nine combinations of route shape (straight, U-shaped, and S-shaped) and participant group (controls, PCA, and tAD), a four-level linear mixed model was fitted that included random effects for participant, route condition within participant (here, the four possible combinations of cue and lighting conditions), and journey within route condition within participant (distinguishing between forward and backward journeys). This allowed for the complex correlation structure of the data. Outliers with long step times were defined as observations with a standardized residual greater than 3; these outliers were dropped, and the model was refitted. Outlier removal was repeated until no further outliers were identified. IMU data were unavailable for 1 trial in one participant (PCA), 2 trials in three participants (1 control and 2 PCA), and 4 trials in one participant (control) owing to recording errors; in addition, IMU data were not analyzed for the participant (tAD) who performed only trials in the absence of the cue to aid in the comparison of outliers between cue conditions.

A dead reckoning technique produced the walking paths and was used to localize outlying (long) step times [33]. For this, all participants were assumed to begin in the same position at the start of the route, and displacement measures were used to calculate the final position and route taken, which were presented visually showing where the hesitant long step times occurred. It should be noted that using dead reckoning to produce these figures provides a measure of resultant end position relative to the start position, rather than an absolute measure of position.

## 3. Results

**Figure 2:**
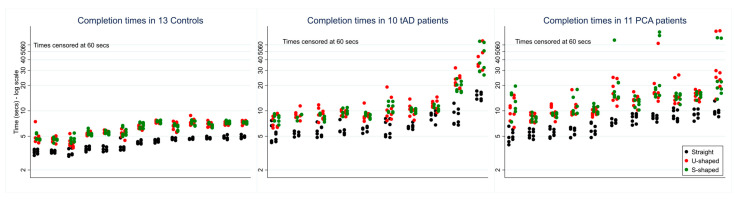
Scatter plots of observed completion time for each participant group under different route conditions (straight, U-shaped, and S-shaped). Plots show individual completion times per participant per trial regardless of other conditions (lighting, cue, and direction). Patients within each group are ranked left to right in order of mean completion time.

Some patient participants were unable to complete the task within 60 s in a small number of trials (a total of 8 trials across 3 PCA patients; 3 trials for a single tAD patient). Owing to the rarity of these uncompleted trials, no formal statistical analysis of accuracy was performed. See Figure 2 for individual completion times for each participant, including a display of the number of trials censored at 60 s. Typically, patients took around twice as long to complete trials compared to controls.

### 3.1. Completion Time

See Table 1 for effects of lighting, cue, and route complexity within each participant group (A) and for comparisons of these effects between participant groups (B). There was no evidence of an effect of lighting on completion time within any group, nor of pairwise differences between groups. The estimated ratios of completion times were all close to 1 as were the ratios of these ratios.

There was some evidence (*p* = 0.047) of an adverse effect of the cue on completion times within the PCA group. The ratio of completion times was 1.126 (95% CI [1.002, 1.267%]) meaning that PCA participants took 12.6% (95% CI [0.2%, 26.7%]) longer to reach the end of the routes in the presence of the dynamic cue. There was no such evidence of an effect of the cue within either the tAD or control groups; in addition, the ratio of ratios comparison was formally statistically significant for the comparison of PCA with tAD (*p* = 0.047) and close to being so (*p* = 0.071) for the comparison of PCA with controls.

### 3.2. Effect of Route

As was to be expected, all groups took longer to complete trials under complex route conditions (S-shaped/U-shaped) relative to the simpler and shorter straight-route condition. However, the relative increases were smaller in controls than in the PCA group (Table 1A). For the U-shaped route compared to the straight route, the estimated ratios of completion times were 1.470 (i.e., a 47% increase) in controls, but 1.914 in the PCA participants, with the ratio of these two within-group between-route ratios being 1.303 (95% CI [1.135, 1.495], *p* = 0.001) (Table 1B). For the S-shaped route compared to the straight route, the analogous ratio of these two between-route ratios was 1.319 (95% CI [1.121, 1.553], *p* = 0.003). Results for the tAD group were intermediate between those for the PCA and control groups, but not formally statistically significantly different from either. There was no evidence that completion time differed between S- and U-shaped routes in any group, with ratios of completion times for these two routes being close to 1 in all three groups; there was also no evidence that these ratios differed between groups.

### 3.3. Kinematic Assessment

**Figure 3:**
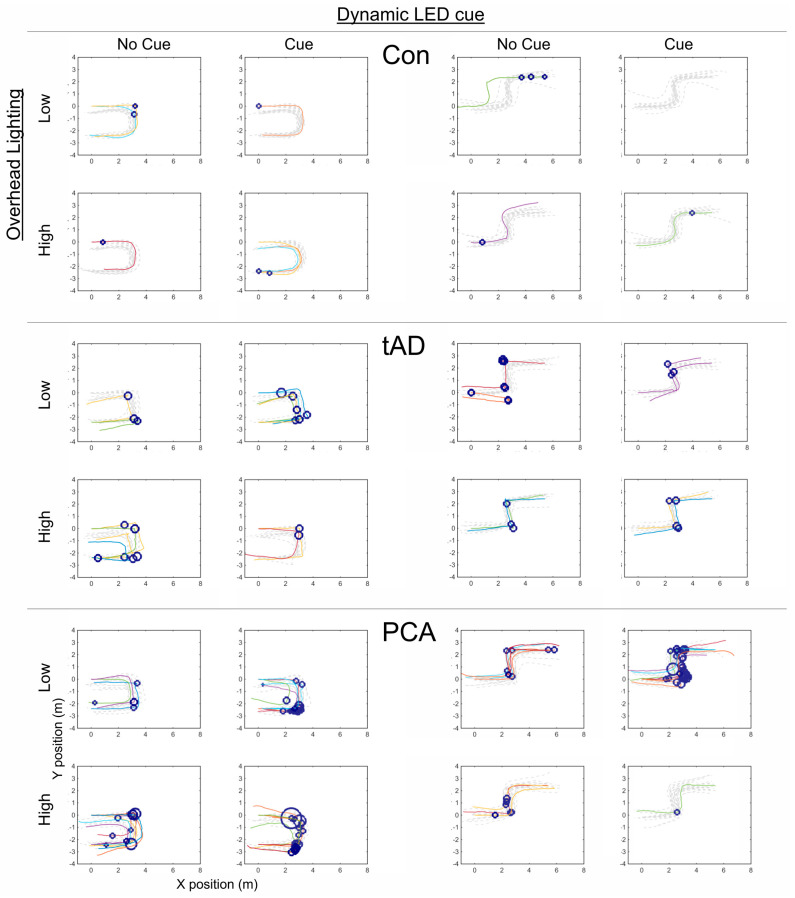
Walking paths with step-time outliers. Displayed for control, tAD, and PCA groups for U- and S-shaped routes under dynamic LED cue conditions (no cue and cue) by overhead lighting conditions (high and low). Dotted lines indicate trials without detected step-time outliers. Step-time outliers are localized using markers, with greater marker size corresponding to longer step times.

Walking paths with step-time outliers are presented in Figure 3 for the control, tAD, and PCA groups for different cue and lighting conditions.

Qualitatively, an increased proportion of outliers were observed in some PCA and tAD patients as they approached regions of routes that required navigational decisions and gait adaptation (i.e., bends). Notably, in the small number of erroneous trials, patients became disorientated to the point where they doubled back on themselves, reaching the start rather than the end of each corridor (tAD: U-shape/low lighting/no cue; S-shape/low lighting/no cue; PCA: U-shape/high lighting/cue; S-shape/low lighting/cue).

Averaged across all conditions, the medians of the observed person-specific median step times were longer in the PCA (step time: 0.70 s; IQR [0.66, 0.78]) and tAD (0.76 s [0.64, 0.84]) groups compared to the control group (0.59 s [0.54, 0.63]). Qualitatively, there was an overall tendency towards higher proportions of observed step-time outliers being detected in both patient groups compared to controls (see Table 2), with the exception of tAD relative to control group outliers under high-lighting conditions in the presence of the cue.

## 4. Discussion

The current study evaluated the effects of environmental conditions on the difficulty of following routes in people with PCA and tAD. Environmental conditions comprised route complexity, overhead lighting, and a dynamic visual cue. In the PCA group, there was evidence that increasing the route complexity approximately doubled the time taken to complete the route compared with analogous increases of around 50% in control participants, with increases for the tAD group being intermediate between these two. However, contrary to our hypotheses, there was no evidence that the higher lighting levels facilitated task performance in either patient group. Additionally, not only was there also no benefit of the dynamic visual cue, but there was evidence of an adverse effect, with PCA (but not tAD or control) participants taking longer overall to follow the routes when a cue was present.

Both the PCA and tAD groups took longer than the controls to complete the routes across all conditions, but there were no marked differences in completion time between the patient groups. Notably, several patient participants were unable to successfully complete a number of trials (unique participants: tAD n = 1; PCA n = 4). This was due to participants taking longer than the 60 s cut off or becoming disorientated and returning to the start point despite the relatively limited task demands involved in walking along closed routes.

Kinematic assessment offers a means to visualize walking paths and outlying steps indicative of a hesitant gait response. Hesitant steps in patient groups detected using kinematic measures were qualitatively observed near locations requiring turning (bends). Poor anticipation of turns might relate to diminished visual processing and visuomotor function, in addition to deficits in spatial memory and executive function undermining predictions of route layout. Previous recommendations regarding the optimal corridor structure for people with dementia are mixed [21,34]. The exaggerated effects of route complexity in both patient groups relative to controls may relate to cognitive demands regarding estimating heading direction, planning turning behavior, and/or varying visual access to the destination [23]. Visual access to destinations while within corridors is now a core feature of dementia-friendly designs, though it is not always feasible given the required structural changes.

There was no evidence of beneficial effects of increased overhead lighting on task completion time in any participant group. A lack of evidence is perhaps counterintuitive, given guidelines suggesting the importance of high levels of lighting within environments designed for people with dementia [34] as well as those suggesting that as much as 50% more lighting should be provided for people with visual impairment [35].

There is recent evidence that lighting variability is associated with slower and hesitant walking in mild PCA [36]. In the current study, it is possible that beneficial effects of higher lighting levels on task performance were confounded by related increases in lighting variability and shadows. Despite comparable numbers of hesitant steps for low- and high-lighting conditions for patients in the absence of the dynamic cue, such hesitations may arise for different reasons. People with PCA often report difficulties in perceiving shadows: they might mistake them for a hole or slope, and this causes hesitation and anxiety when navigating areas with high contrast and dark shadows [37]. Similar reports relate to people with tAD at a later stage [38]. Studies have reported people misperceiving their own shadows as unequal flooring and stepping over them, concluding that dark lines could cause anxiety or disorientation [39]. Guidelines do suggest that areas of high-contrast shadows should be avoided and that environments should prioritize uniform lighting, high contrast, and reduced glare [22]. Future investigations might jointly evaluate the effects of overall overhead lighting levels, uniformity of lighting, and flooring contrast on patient function.

Contradicting our hypothesized benefits of the dynamic visual LED cue, there was some evidence that the PCA group took longer to reach the destinations with the cue present. Adverse effects of the dynamic cue were particularly evident in the PCA group relative to the tAD and control groups, neither of which exhibited any significant cue effect. This cue was designed to appeal to the relatively spared abilities of people with PCA and tAD reported by previous studies: specifically, residual visual processing of moving relative to static stimuli and fixation of visually salient features within scenes [25,26,40]. Notably, while previous studies were carried out with patients who were seated in an essentially stationary position, the current task required walking to destinations in directions congruent with the dynamic cue. It may be that discrepancies between participants’ walking speed and dynamic cue rate were disruptive to optic flow perception, an important cue for navigation and self-motion [41]. Adverse cue effects might also be explained by a deficit in visually disengaging from the salient cue, at the expense of successfully and efficiently completing the task. Previous studies have found a deficit of disengagement of visuospatial selective attention in people with tAD and PCA [5,12,42]. Such deficits in disengaging attention or fixation are characteristic of Balint’s syndrome and are core features of PCA. This disengagement deficit has been ascribed to superior parietal dysfunction, an area in which PCA groups have been found to have particular reductions in grey matter volume and lower cortical thickness compared to people with tAD [7,43]. The cue is also similar to adaptations that are already being utilized in the homes of people with PCA, emphasizing a need for formal evaluation of adaptations to better inform recommendations.

Future work to identify cues to support navigation might explore whether the current visual paradigm can be usefully adapted (i.e., by using a single moving target or different motion patterns) and/or combined with strategically placed visual cues. There is some evidence that landmarks and visual cues may offer a means to aid navigation [14], particularly where direct visual access is not possible. Nonvisual sensory cues such as tactile paving may be a worthwhile focus of future investigations given recent evidence of increased effects of haptic cues on orientation in PCA and tAD [44]. While signage has been proposed to aid navigation for people with dementia in residential settings, it is important to consider the signage used (i.e., pictorial or written) and also its positioning (i.e., lower than eye level for people with advanced dementia who tend to look at their feet when walking [15]).

Strengths of this study include a repeated-measures design with a high number of trials, counterbalanced within and between participants to control for order effects. Participants are well characterized regarding their clinical and neuropsychological profile and measures of completion time and gait. Limitations of this study include a relatively small and heterogenous patient sample owing to the rarity of PCA, and formal a priori power calculations were not calculated, partly owing to challenges in estimating effect sizes in this first-of-its-kind study and partly due to the difficulty of performing sample sizes for this complex, repeated-measures design with little information about the various parameters (means, variances, and correlation coefficients) that would be needed in order to perform the necessary calculations. Standard measures of mobility (e.g., the Barthel index) were not administered but could be in future studies to determine the correspondence between performance on the reported experimental task and commonly used clinical metrics. Furthermore, this study took place in a laboratory setting enabling control of particular aspects of the environment (i.e., lighting, layout, and prior familiarity), and so caution must be exercised in generalizing results to familiar home environments. However, the current dead reckoning and outlier detection techniques enable assessment without requiring laboratory infrastructure and may be used to extend and replicate findings in community and long-term care settings.

## 5. Conclusions

This study evaluated route following in people with PCA and tAD under different environmental conditions. The findings emphasize the particularly inefficient task performance in patient groups under more complex route conditions. Despite previous reports of the benefits of signage and increased lighting on navigation in dementia [23], there was an adverse effect of a dynamic visual cue on route following in the PCA group and no significant effects of overall lighting levels in any participant group. Future directions should investigate alternative environmental adaptations to aid navigation, the effects of uniformity and degree of overhead lighting, and the optimal positioning of any cues or lighting features along sections of different routes.

## Figures and Tables

**Table 1:** Estimated completion times expressed as (**A**) between-condition ratios comparing lighting, cue, and route conditions within each participant group and (**B**) ratios comparing lighting, cue, and route ratios between participant groups (i.e., ratios of ratios). 95% CIs are in brackets.

**A**	**Controls**	**PCA**	**tAD**
Light high/low	0.980 (0.942, 1.019)	0.983 (0.871, 1.109)	0.945 (0.822, 1.087)
Cue/no cue	1.012 (0.997, 1.028)	1.126 (1.002, 1.267)	0.995 (0.947, 1.044)
Route U/straight	1.470 (1.409, 1.533)	1.914 (1.673, 2.189)	1.741 (1.467, 2.065)
Route S/straight	1.491 (1.443, 1.542)	1.968 (1.675, 2.312)	1.690 (1.453, 1.967)
Route S/Route U	1.015 (0.991, 1.039)	1.028 (0.950, 1.112)	0.971 (0.926, 1.019)
**B**	**Controls**	**PCA**	**tAD**
Light high/low	1.003 (0.885, 1.136)	0.965 (0.837, 1.113)	0.962 (0.810, 1.143)
Cue/no cue	1.113 (0.989, 1.252)	0.983 (0.935, 1.033)	0.883 (0.781, 0.998)
Route U/straight	1.303 (1.135, 1.495)	1.185 (0.966, 1.409)	0.909 (0.742, 1.115)
Route S/straight	1.319 (1.121, 1.553)	1.134 (0.972, 1.321)	0.859 (0.699, 1.056)
Route S/Route U	1.013 (0.935, 1.098)	0.957 (0.909, 1.007)	0.945 (0.866, 1.031)

**Table 2:** Estimated proportions of step-time outliers for different lighting conditions for PCA, tAD, and control groups, averaged across all straight/S-/U-shaped conditions across both trial directions (forwards/backwards).

	Controls		PCA		tAD	
	No Cue	Cue	No Cue	Cue	No Cue	Cue
Low lighting	1.14%	0.43%	1.41%	4.54%	1.52%	1.04%
	8/703	3/704	15/1,067	56/1,235	14/923	9/862
High lighting	1.05%	1.29%	1.59%	2.22%	1.17%	0.71%
	7/669	9/699	18/1131	25/1128	10/852	6/840

## Data Availability

Data and analytic methods are available via https://reshare.ukdataservice.ac.uk/853147/.

## Supplementary Materials

The following supporting information can be downloaded at https://www.mdpi.com/article/10.3390/brainsci14121217/s1: Table S1: patient demographic information and neuropsychology assessments. Patients within each group are ranked left to right in order of decreasing time to complete straight routes, averaging over lighting and cue conditions. Bold scores indicate performance below the 5th percentile based on normative data. References [45,46,47,48,49,50,51,52] are cited in the supplementary materials.
