# Supplementary material for: Effects of Route Complexity and Lighting on Route Following in Alzheimer’s Disease and Posterior Cortical Atrophy
Source: Brain Sci. 2024 Nov 30;14(12):1217. doi: 10.3390/brainsci14121217 (PMC11726922; doi:10.3390/brainsci14121217)
Supplement: Supplementary file 1 [file brainsci-14-01217-s001.zip › brainsci-3281081-supplementary.pdf]

Supplementary Table S1: patient demographic information and neuropsychology assessments. Patients within each group are ranked left to right in order of decreasing time to complete straight-routes, averaging over lighting and cue conditions. Bold scores indicate performance below the 5th%ile based on normative data.

		**Patients (ordered L to R by decreasing completion time on straight route trials)**																					
	**Patient number**	1	2	3	4	5	6	7	8	9	10	11	12	13	14	15	16	17	18	19	20	21	
	Diagnosis*	TAD	TAD	TAD	TAD	TAD	TAD	TAD	TAD	TAD	TAD	PCA	PCA	PCA	PCA	PCA	PCA	PCA	PCA	PCA	PCA	PCA	
	Age (years)	69.4	66.0	67.5	69.1	74.4	64.0	59.1	59.1	70.9	62.7	68.3	75.0	66.0	67.2	57.2	60.2	64.4	70.6	70.6	57.9	58.9	
	Gender	M	F	F	F	M	M	F	F	M	F	F	M	F	F	F	M	M	F	M	F	M	
	MMSE (/30)	14	25	15	26	23	13	15	18	22	15	23	28	22	21	22	10	15	12	16	22	11	
		**Background neuropsychological assessment**																					Normative mean (SD)
General	sRMT words (/25) [46]	**12.5**	23	**18**	**18**	**15**	**18**	18	**14**	**12.5**	**15**	23	21	23	21	**18**	**12.5**	21	**12.5**	23	**13**	**12.5**	23.7 (1.8)
	Concrete synonyms (/25) [47]	**14**	19	**17**	24	22	20	**18**	24	22	19	23	21	24	19	23	**18**	19	**18**	**16**	23	19	23.0 (2.5)
	DS forwards (/12)	**4**	8	7	8	7	**4**	5	7	6	5	7	10	4	6	7	**2**	**0**	4	**1**	6	**2**	7.5 (2.0)
	DS backwards (/12)	**2**	4	3	4	4	**2**	**2**	**2**	5	3	4	6	4	**2**	6	**0**	**0**	3	**2**	**0**	**0**	6.3 (2.2)
Non-	Calculation (/24) [48]	**0**	4	**0**	10	14	**0**	**0**	**0**	**0**	**0**	**0**	9	**0**	**1**	**1**	**0**	**0**	**0**	**2**	**0**	**0**	12.0 (5.1)
	Spelling (/20) [49]	**4**	19	6	18	7	12	11	8	8	20	12	16	7	6	17	**1**	**0**	**1**	**3**	16	6	19.5 (6.5)
	Gesture production (/15)	15	15	9	15	11	13	15	15	15	10	12	13	15	12	10	11	3	8	14	15	4	-
Early visual	Visual Acuity	6/18	6/9	6/18	6/9	6/9	6/9	6/9	6/9	6/9	6/9	6/9	6/9	6/9	6/12	6/9	6/12	6/12	6/9	6/18	6/24	6/24	-
	Crowding (/10)	10	10	10	10	10	10	10	10	10	**4**	**0**	**9**	10	10	10	**4**	**2**	**0**	10	**6**	**0**	10
	Figure ground (/20) [50]	19	20	19	20	**18**	**17**	**15**	**18**	20	**15**	**10**	**15**	**17**	**11**	19	**10**	**10**	**10**	**17**	**18**	**10**	19.9 (0.3)
	Shape Discrimination (/20) [51]	**19**	20	20	20	20	20	**10**	**15**	20	**9**	**13**	**15**	20	**15**	**18**	**17**	**8**	**4**	**17**	20	**10**	20
Visuo-spatial	Number location(/10) [50]	**0**	9	**0**	10	9	**5**	**2**	**0**	9	**0**	**0**	**6**	7	**4**	**4**	**0**	**0**	**0**	8	**0**	**0**	9.4 (1.1)
	Dot counting(/10) [50]	10	10	10	10	10	**7**	**0**	**6**	10	**3**	**0**	**0**	10	**6**	**6**	**0**	**0**	**0**	10	**4**	**0**	-
	A cancellation (max 90s) [52]	45	24	**41**	**40**	28	**39**	**59**	**73**	**56**	**90**	**90**	**72**	**90**	**71**	**59**	**90**	**90**	**90**	**33**	**79**	**78**	20.5s (6.5)
	Letters missed	**0**	**0**	**0**	**0**	**0**	**2**	**2**	**0**	**1**	**6**	**8**	**5**	**0**	**2**	**1**	**19**	**19**	**15**	**1**	**1**	**10**	**-**
Visuo-	Object decision(/20) [50]	**10**	17	17	18	19	19	16	17	20	**5**	**5**	15	17	**11**	**13**	**4**	**11**	**2**	15	**6**	**3**	17.7 (1.9)
	Fragmented letters(/20) [50]	17	19	**15**	18	20	20	**0**	**14**	19	**2**	**0**	**6**	**14**	**0**	**10**	**0**	**0**	**0**	**13**	**3**	**0**	18.8 (1.4)
	Unusual views (/20) [53]	**9**	**10**	**9**	13	15	20	**10**	15	17	**0**	**0**	**3**	**3**	**0**	**1**	**-**	**6**	**-**	13	**0**	**-**	17.1 (3.0)
	Usual Views (/20) [53]	**19**	**18**	20	20	**19**	20	20	20	20	**8**	**0**	**16**	**17**	**11**	**12**	**-**	**19**	**-**	20	**8**	**-**	19.7 (0.5)
